# Who Are Dentists?

**Published:** 1861-05

**Authors:** Wm. A. Pease


### WHO ARE DENSISTS ?—No 3.

BY WM. A. PEASE.

THE question will frequently arise in the mind of persons about getting a set of artificial teeth, " which of the various methods by which they are inserted, and which of the different materials on which they can be mounted as a base will be the best, the most comfoitable, durable and natural; and which will prove the most satisfactory ?" As such persons wish to act intelligently, and as it is frequently with them a subject of consideration and consultation with their friends as well as with dentists, it is proposed in this article to state clearly and briefly the advantages and disadvantages of the different methods of inserting artificial teeth, and the comparative value of the different materi ls or bases upon which they are mounted. It should be borne in mind, however, that the estimate here given is not absolute, but relative; it applies to the general run of cases, and is he rule to which it will be the safest to adhere; although there may be exceptions requiring different methods or other materials; but, if they occur, they will be of such a nature that, when explained, they will be easily understood by the person, who will see from the shape or condition of the mouth the reason and necessity of the departure from the usual method or material. At this time it is believed that such a statement will furnish valuable data to two classes of persons, viz: those who have lost their teeth, and those whose teeth are decayed, and who are balancing in their mind the propriety of having them plugged, or of allowing them to decay and break off with the view of getting artificial ones.

To fully understand the reason why different operators employ different methods of inserting artificial teeth, and why there are so many different and widely dissimilar materials used as a base for that purpose, a few preliminary remarks will be necessary.

The country is now full of dental mechanics; and the facility with which they are manufactured in the laboratory, and the mistaken idea that dentistry affords lucrative employment is fast increasing the number. Men are wanted in all the principal offices in the country to do the office drudgery and to make artificial sets of teeth. Hence, apprentices are taken who, after serving their time, are dismissed or employed at mechanics' wages; they are not called dentists by the profession. These men are often ambitious; having seen some operations in the mouth while in the office; having no professional reputation at stake or character to lose, they boldly assume to be dentists and boldly essay to perform all operations at the chair as well as to make mechanical work. Conscious of their inability to operate as well as dentists, and satisfied with mechanics' wages, they endeavor to gain business by all the tricks and arts of trade, and by placing the price of their work so low, that no dentist can compete with them and do justice to his patients, maintain his character as an honest man, or his position in the profession. Notwithstanding all these advantages they know that they can not long sustain themselves in competition with dentists by these means alone; they must resort to other means and other devices to engage the public attention and divert it from their unsuccessful operations. Hence they eagerly seize upon every new material, instrument, device or mode of practice, however unpromising it may be, and thrust it upon the public attention, until it has had its run, its worthlessness is discovered, and the public are disgusted with them and with dentistry in general.

Thus it will be seen that but for them many of these materials and modes of practice would have been little known outside of a few dental offices, where they would quietly have been subjected to the severest tests of experiment and experience until their worthlessness was discovered. The confidence of the public would not then have been abused, dentists would not have suffered from the mortifying failures, and the public would have been better served. And here I will remark the public should view with distrust all new materials and improvements unless sanctioned by men of known probity and character, and even then people had better not lightly depart from the proved methods and materials, as the best of dentists may be mistaken.

The methods by which artificial teeth are now inserted upon metallic or other bases are two, viz.: either by springs or clasps, or atmospheric pressure. Those attached to the natural teeth by means of clasps are partial sets consisting of a variable number of teeth, and they are attached in this way for various reasons, the principal of which are: first, price; they can be inserted cheaper by clasps than by atmospheric pressure. Second, the peculiar shape of the mouth, or the peculiar way in which the teeth of the upper and under jaw strike or come together, which sometimes make a partial set by suction impracticable. When a set of teeth is inserted by clasps, the plate covers much less of the roof of the mouth than it does by atmospheric pressure, and it is generally firmer and more serviceable in eating; but there is a serious objection to it, as it causes a rapid decay of the natural teeth to which the set is attached.

While in some mouths the teeth are so healthy that they can withstand almost any kind of abuse, in others they are so unhealthy that they can be preserved with the greatest difficulty. Unfortunately it is the latter class that generally require artificial teeth; and it is found by experience that the teeth, to which the clasps are attached, will generally require to be plugged in from one to two years after the set has been inserted; and they will frequently break off in one or two years more, so that after four or five years the person has not only lost the artificial teeth, but also two of the best natural ones. This may be considered an average in young persons, for while some sets are lost in a much shorter time, others will be more durable. On the contrary, a suction set does not injure the natural teeth, and if it consists of several teeth and they are continuous, if the remaining natural teeth are ultimately lost, its adaptation or value is not materially lessened. But it should be borne in mind that it is a greater incumbrance to the mouth, and generally it is not as useful in masticating the food. These are the practical advantages and disadvantages of the two methods of inserting artificial teeth.

Coming now to the different materials which can be used for a base for artificial teeth; they will be found to be divided between the mineral and vegetable kingdoms. In the mineral they are gold, silver and platina. In the vegetable they are the gums of the gutta percha and India rubber trees. It is not surprising that a metal so beautiful as gold, so incorruptible and plastic, and one, withal, of so great intrinsic value should be considered almost instinctively by the profession and the community as one eminently suitable to be a base for artificial teeth. Alloyed and adulterated as it often has been until it was sometimes difficult to determine whether it predominated over a mixture of silver and copper; it has, nevertheless, maintained its position in the professional and popular estimation; and it is believed that single gum-teeth well mounted upon good gold plate make the best practical artificial denture that has yet been devised. The reason of this is obvious; there is no metal so good as gold, and none, like it, have stood the test of time and experience; and there is none that can be used for partial sets, that harmonize so well and produce so little galvanic disturbance when brought into contact with plugs in the natural teeth. Passing over silver which is chiefly valuable for temporary purposes, we come next to platina, a metal that has come into use within the last ten years as a base for enameled or continuous gum work, (so called because the gum is fused into one piece,) and it is only used for that purpose, because it is the only metal fit to remain in the mouth, that does not melt at a less heat than the enamel that is to be fused upon it to form the gum. The question will then arise, what are the advantages and disadvantages of a set of teeth made with continuous gum over one made in the ordinary manner on gold plate? We answer the only advantage (which is of some moment,) consists in melting the enamel between and around the teeth so as to leave no space for the accumulation of food; and in some few instances, of building out beyond the plate, so as to give the mouth and cheeks a little more fullness. Having stated all of the advantages of continuous gum work over ordinary sets of teeth on gold, let us see if there are any disadvantages which neutralize them and make it less desirable as a whole; for the public have a right to know if such there are. All suction sets of teeth are held in their place by traction upon the roof of the mouth. In other words, the air is drawn from between the plate and the gum, or mucous membrane of the roof of the mouth, when this pliable membrane closes around the edges and draws into the chamber of the plate to prevent the air from re-entering. Thus it will be seen that the weight of the set of teeth and the pressure upon it during mastication have to be sustained by the tender mucous membrane of the roof of the mouth; and is to that extent, a constant and unnatural tax upon it during life. Hence, the lighter the material the less is the strain or tax, and the greater must be the force during mastication to overcome the adaptibility of the mucous membrane to the plate and loosen the set; and hence, that material which has strength with the least bulk, which is cleanly, inoffensive, and has nothing incompatible with the health or well-being of the mouth, is the best adapted for dental purposes, and should always be used. Platina has not these qualities to so great an extent as gold. It is a heavier and softer metal than that quality of gold used to make artificial dentures; and hence, to get the necessary strength it must be used thicker than gold; or else, the plate must be strengthened by bands or stays, and by the time the enamel is fused upon it, the set is thicker, more clumsy and heavier than a set of teeth of equal strength mounted upon gold. Nor are these the only objections to platina as a base for artificial teeth. One of the chief duties of a dentist in constructing an artificial denture is to provide for repair. This is too much lost sight of by the profession, and is almost wholly overlooked by the public, who seem to think that, having obtained a set of teeth, it will last through good and bad usage for life; whereas, nothing is more fallacious, and nothing tends so much to make them neglect the natural teeth.

A set of artificial teeth is a piece of mechanism—nothing more. It is made of light, frail and fracturable materials, and it is believed that the average duration of sets of artificial teeth will not exceed six years, while many of them are a constant source of trouble and expense from the first. From the nature of the case, a set of teeth made to fit a changeable base must be perishable. The human mouth is constantly undergoing changes; small it is true, but appreciable, when measured by an unchangeable set of teeth. Hence, a set of teeth not entirely inelastic, one that will spring and accommodate itself a little to the changes of the mouth, will be the most comfortable and durable, and fulfill more of the requirements of a good denture than one of greater rigidity. These qualities are possessed by gold and silver, but they are not possessed by continuous gum or enameled work; for, the enamel being vitrous, or like glass, is inflexible, and if the platina base springs, however little, the body or enamel checks, cracks or flakes off. Hence, a set of this kind is more liable to accident from a fall or a change in the shape of the mouth, and if there is any warpage, or the set bends, it can not be restored to the original form, like those on gold or silver.

Another important element of value of all mechanical work is universality of manufacture. A piece of mechanism, however valuable it may be, loses much of its value if the means of repair are not always at hand, in case of an accident. Few would carry watches if they had to send them fifty or one hundred miles for repairs; and watches are less liable to accidents than artificial teeth. Hence, it follows that that kind of artificial teeth that can be made, and easily and cheaply repaired by all dentists, not only of a particular locality, but by all dentists of the country, are the safest, and therefore the most desirable and valuable. . Such are those mounted on gold and silver; such are not those mounted on other materials, as they require a peculiar apparatus for their construction and repair, not used for other materials. Besides, all dentists know that persons when abroad are frequently subject to expense and serious inconvenience from some accident to their teeth, which they can not get repaired where they are, because they were made in an unusual manner; and that at home they are liable to the same inconvenience, from the death or removal of their dentist. Thus it will be seen that a set of teeth on a platina base is valuable for cleanliness, and occasionally for other purposes, but that it is undesirable from its weight, clumsiness, greater tax on the roof of the mouth, and also, from its liability to accidents not easily repaired.

(To be continued.)

